# Lower extremity kinematics in children with and without flexible flatfoot: a comparative study

**DOI:** 10.1186/1471-2474-13-31

**Published:** 2012-03-02

**Authors:** Yi-Fen Shih, Chao-Yin Chen, Wen-Yin Chen, Hsiu-Chen Lin

**Affiliations:** 1 Department of Physical Therapy and Assistive Technology, National Yang-Ming University, 155, LiNong Street Section 2, Pei-Tou District, Taipei, Taiwan 112; 2 Department of Physical Therapy, China Medical University, Taichung, Taiwan

## Abstract

**Background:**

A high percentage of young children present with flatfeet. Although the percentage of those with flatfeet declines with age, about 15% of the population maintains a flat arch. A reduction in longitudinal arch height usually combines with excessive subtalar joint pronation and may be related to other musculoskeletal problems of the lower extremity kinetic chain. The purpose of this study is to describe and compare the lower extremity kinematics between children with normal arches and those with flexible flatfeet, with the intent of providing practical information for decision making when treating children with flexible flatfeet.

**Methods:**

Twenty children with flexible flatfeet (years age mean (SD), 9.7 (0.9) years) and 10 children with normal arches (yeas age mean (SD), 9.6 (1.2) years) were included. Kinematic data (maximum and minimum angles, and movement range, velocity, and excursion) of the hip, knee and rearfoot were collected during walking using Liberty Electromagnetic Tracking System. Kinematic variables were compared between the normal arches and flexible flatfeet groups using repeated measures mixed effects ANOVA.

**Results:**

Movement patterns at the hip, knee and ankle joints were similar between children with flexible flatfeet and with normal arches. The results of ANOVA showed no significant main effect or interaction in any of the kinematic variables (P ≥ 0.05).

**Conclusions:**

This study identified no kinematic adaptation during walking in children with flexible flatfoot. We suggested that future research should take the influence of the mid-foot and forefoot into consideration when examining lower extremity kinematics in children with flexible flatfoot.

## Background

Flexible flatfoot is a condition in which the medial longitudinal arch of the foot collapses during weight bearing and restores after removal of body weight [1,2]. Prevalence of flexible flatfoot in children, 2 to 6 years of age, has been reported at between 21% and 57%, and the percentage decreased to 13.4% and 27.6% in primary school children [3-5]. Generally, infants are born with flexible flatfoot [3]. The development of foot arch is rapid between 2 and 6 years of age [6], and becomes structurally matured around 12 or 13 years of age [7].

Many individuals with flexible flatfeet walk with certain alterations in the lower extremity kinematics. The most common alteration is excessive pronation of the subtalar joint during stance phase [4,8-10]. In normal gait, the subtalar joint starts to pronate after initial contact until the metatarsal head contacts the ground, where upon the subtalar joint starts to supinate and converts the foot into a rigid structure for propulsion in the late stance phase [11,12]. In people with flexible flatfeet, the foot stays in a pronated position without turning to supination early enough during the late stance phase [13], which is not efficient for completing the push-off during gait [11,14,15]. Considering the coupling movement between rearfoot inversion/eversion and tibial rotation [16,17], an excessive or prolonged pronation of the foot is often linked to excessive or prolonged tibial rotation and larger valgus at the knee [9].

Although flexible flatfoot in children rarely causes pain or disability, Lin et al. suggested that kinematic changes and the resulting gait deviations may lead to lower extremity pathologies later in life [4]. This rationale supports the use of insoles or corrective shoes for the treatment of children with flatfeet. Despite the available evidence of the effect of flatfoot on lower extremity kinematics in adults, very little was known about how flexible flatfoot impacts children [4]. Therefore, the aim of this study was to describe and compare the lower extremity kinematics in primary school children with and without flexible flatfoot. We hypothesized that children with flexible flatfoot exhibited kinematic alterations for the ankle/foot segment and the knee and hip joints compared to children with normal arches.

## Methods

### Subjects and inclusion/exclusion criteria

A total of 274 children seven to ten years of age from two elementary schools in Taipei, Taiwan were screened for the study. Children with a history of traumatic injuries or surgeries in lower extremities were excluded from this study. Also, children with a clinical diagnosis of developmental delay, current psychological or physical illnesses that would influence normal gait performance or children unable to follow commands were excluded. All eligible children were assessed with the Feiss line method [18]. The Feiss line is an imaginary line which connects the apex of the medial malleolus and the plantar aspect of the first metatarsophalangeal joint. Ideally, the navicular tuberosity lies on or very close to the line. The distance of the Feiss line to the floor can be divided into three equal parts. If the navicular tuberosity falls within the middle one third, it represents a second-degree flatfoot (moderate flatfoot); and if it falls into the bottom third, it presents a third-degree flatfoot (severe flatfoot). Lin and co-workers suggested that Feiss line method was an objective means to define flexible flatfoot as compared to observation of the height of medial longitudinal arch. In addition, this test required no need of identifying subtalar neutral position, which may be harder to execute and not reliable enough in young children [4]. We recruited only subjects with second and third-degree flatfeet (N = 107). Parents were then provided information about the study and were asked to select from the following choices: (1) I am interested in participating in the study; (2) I would like more information about the study before deciding; (3) I am not interested in participating. Of those agreeing to participate in the study, twenty children with bilateral flexible flatfeet volunteered to participate. Ten age-matched children without flatfeet were recruited and served as the comparison group. Children and their parents were informed of the purpose and procedures of this study and children provided assent and parents provided informed consent to participate. All procedures were approved by the Institutional Review Board of National Yang Ming University, Taipei, Taiwan (IRB number: 970020R).

### Measurements

Assessment of lower extremity alignment was performed before walking trials. The measurement included weight-bearing and non-weight bearing calcaneal eversion angle, navicular height, quadriceps (Q) angle, ankle flexibility (ankle range of motion with knee flexion and with knee extension), and femoral anteversion [18].

**Figure 1:**
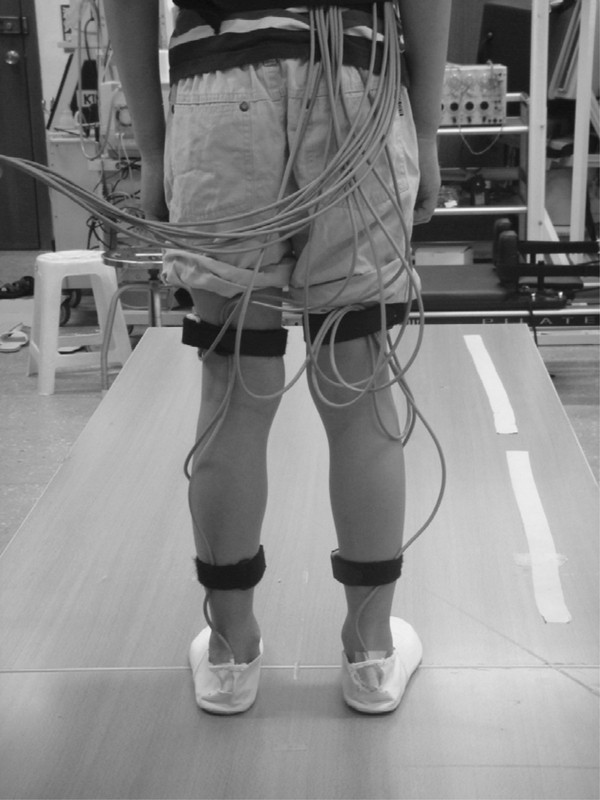
**Illustration of subject preparation for the walking test**. One motion sensor is on the center of sacrum, and six other sensors are placed bilaterally at lower third of the lateral thigh, lower third of the medial shin, and the posterior surface of the calcaneus lateral to the insertion of Achilles tendon.

The kinematic data of the lower extremities during walking were collected at 240 Hz using LIBERTY electromagnetic tracking system (Liberty Polhemus, Colchester, VT, USA). The transmitter, the source of the electromagnetic field, was anchored on a tripod, and placed 40 cm above an elevated wooden walkway. This setup minimizes the interference of the electromagnetic field from the ground steels. A pen-like stylus was used to digitize the anatomic bony landmarks for defining the segmental coordinate system [19]. Five standard-sized sensors were used to record the movement of the pelvis, and bilateral thighs and shanks, and two small sensors were used to record movement of the calcaneus. These sensors were placed at the center of the sacrum, lower third of the lateral thigh over the iliotibial band, lower third of the medial tibial bone and posterior surface of the calcaneus lateral to the insertion of Achilles tendon (Figure 1). These sensors were secured with elastic tape and reinforced with an elastic strap in order to reduce extraneous movements on the skin during walking.

Subjects were provided with a pair of indoor shoes for testing. The test started with digitization of the bony landmarks in the relaxed standing position, with toes directed forward and feet separated about a shoulder width apart. The digitization procedure provided information to transform sensor orientation data into the local anatomic coordinate systems for description of relative movement between segments of the thigh, lower leg and calcaneus. A total of sixteen bony landmarks were used, adapted from the recommendations of International Society of Biomechanics (ISB), including both sides of anterior/posterior superior iliac spine, medial/lateral femoral epicondyle, medial/lateral tibial condyle and medial/lateral malleolus [19]. After digitization, the subjects were instructed to perform three repetitions of hip flexion/extension and hip abduction/adduction with each leg in order to define the center of the hip. Then, the child was told to walk naturally 10 times back and forth on the walkway while the kinematic data were collected. The kinematic data from one complete gait cycle was selected from each walking trial for further analyses.

The kinematic data were calculated based on the recommendations from ISB [19] and from Grood and Suntay [20] using a self-written C++ based program. Because the hip joint center could not be digitized directly, a regression analysis method was used to estimate the joint center position while the subjects were performing hip flexion/extension and abduction/adduction. The rotational motions of each segment were presented with respect to the adjoining proximal segment [21]. Rotational sequences and axis definitions were as followed: first rotation about the X-axis which was pointing medial-to-lateral, then about the Z-axis which was the floating axis perpendicular to both X- and Y-axes, and last about the Y-axis which was pointing distal to proximal for the right side and the opposite for the left side [21]. The occurrence of the maximum superior acceleration of the calcaneal sensor marked the event of foot contact. A gait cycle was the period between two events of same foot contact, and the first 60% of the gait cycle was defined as the stance phase [22-24].

The kinematic variables including the joint rotation angle at initial contact, as well as the maximum and minimum angles, and the movement range of the calcaneus, knee joint and hip joint rotation in sagittal, frontal, and transverse planes were extracted from the stance phase. In addition, the excursion, defined as the range from initial contact to the appearance of maximum angle, of the calcaneal eversion and the internal rotation of the knee and hip, was also calculated. Researchers suggested that the joint excursion information contained important parameters describing the flatfoot influence on the movement of the lower extremity kinematic chain [16,25]. Our method of kinematic measurement has been used in adults [23], but not in children between 7-10 years old. However, similar joint kinematic calculation has been reported among children [24], and data from Ganley et al. suggested that children at the age of 7 years showed similar anthropometric proportions as compared to adults [25]. The measurement repeatability of lower extremity kinematics using an electromagnetic system has been established previously [23]. Our pilot study revealed good to excellent between-session reliability (ICC = 0.76 to 0.95) of the lower extremity kinematics measurement except an ICC of 0.62 for the maximum calcaneal eversion angle. The standard error of measurement (SEM) was 1.51° to 2.17° for the maximum angle of segmental rotation, 1.05° to 1.5° for the joint rotational excursion. Because the averaged data from 3 walking trials resulted in best measurement repeatability and similar movement patterns of bilateral legs were observed in the pilot study, the averaged kinematic data of the right limb from 3 stance phases were extracted for statistical analyses.

### Statistical analysis

The repeated measures mixed effect analysis of variance (ANOVA) was used to compare the kinematic variables. The within subject factors were joint (hip, knee, and subtalar joints), plane of movement (sagittal, frontal, and horizontal planes), and various points across the stance phases. The between subject factor was group (flexible flatfoot vs. normal group). Group differences were expressed as mean difference [95% CI (confidence interval)]. Statistical analyses were performed with the Statistical Package for Social Sciences version 12.0 (SPSS for Windows release 12.0, Chicago, USA). The level of significance was set at *P *< 0.05.

## Results

### Subject description

Twenty children with flexible flatfeet (mean age (SD), 9.7 (0.9) years) and 10 children with normal foot arches (mean age (SD), 9.6 (1.2) years) were included. Demographic characteristics of the participants are summarized in Table 1. Despite an unequal sample size, there was no significant group difference in age, height, weight, body mass index, and measurement of Q angle, femoral anteversion, and ankle flexibility. The measurement of the flexible flatfoot, the weight-bearing calcaneal eversion angle and navicular height differed between the two groups, showing that foot arches collapsed more in children of the flexible flatfoot group (Table 1).

**Table 1:** Basic characteristics and lower extremity alignment measurement in children with and without flexible flatfoot

	Flexible flatfoot group, N = 20 Mean (SD)	Control group, N = 10 Mean (SD)	Mean Difference (95% CI)
Age (years)	9.7 (0.9)	9.6(1.2)	0.17 (-1.01 to 1.35)
Height(cm)	133.53 (7.61)	137.2 (8.26)	1.6 (-4.99 to 8.12)
Weight(kg)	30.23 (6.18)	31.8 (5.54)	4.0 (-4.14 to 12.09)
Body mass index (kg/m^2^)	16.8 (2.2)	16.82 (2.14)	1.1 (-2.34 to 2.35)

Non-weight bearing Calcaneal eversion/inversion (°)^a^	-0.75 (4.43)	-0.3 (4.57)	0.45 (-3.10 to 4.00)
Weight bearing calcaneal evrsion/inversion (°)^b^	10.03 (3.18)	5.95 (1.38)	**-4.08 (-5.79 to -2.37)**
Weight bearing navicular height (cm)	3.17 (0.91)	4.13 (0.27)	**0.96 (0.35 to 1.56)**
Q angle (°)	16.25 (2.37)	17.6 (7.78)	-1.35 (-6.55 to 3.85)
Ankle ROM with knee extension (°)	16.13 (12.34)	15.85 (9.11)	-0.28 (-9.32 to 8.77)
Ankle ROM with knee flexion (°)	35.48 (17.92)	29.95 (13.11)	-5.53 (-17.43 to 6.38)
Femoral anteversion (°)	10.15 (4.45)	9.9 (3.09)	-0.25 (-3.47 to 2.97)

^a^: +:calcaneus eversion; -:calcaneus inversion

^b^: +:calcaneus eversion; -:calcaneus inversion

95%CI: 95% Confidence interval of the difference

Statistically significant associations are given in bold face

### Lower extremity kinematics

**Figure 2:**
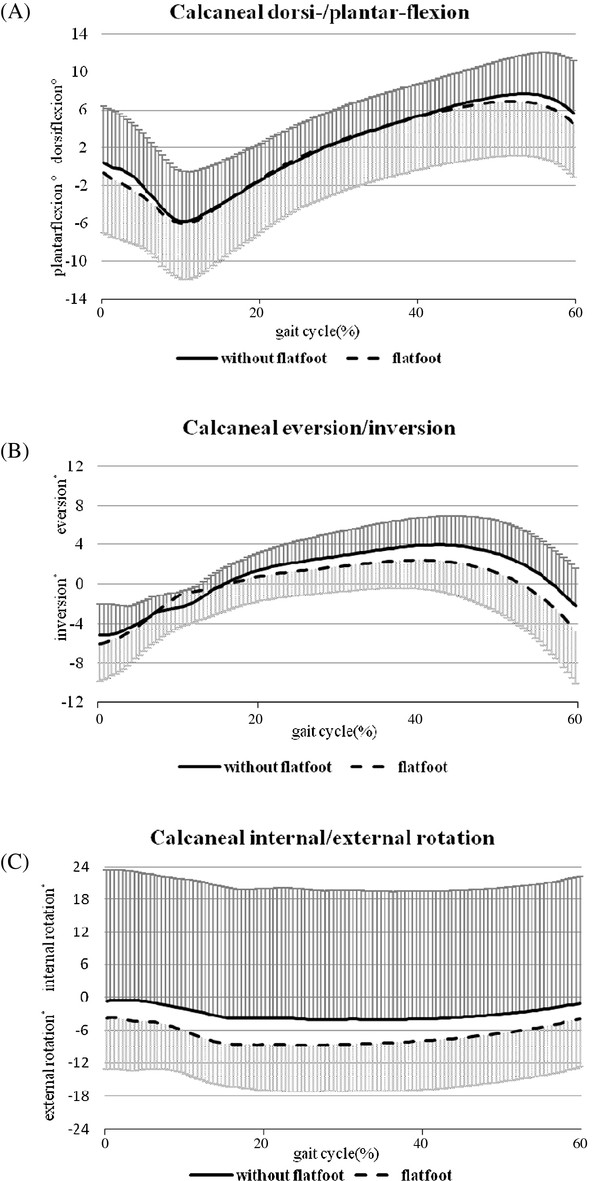
**Movement pattern of ankle/foot complex**. (A) Calcaneal dorsi-/plantarflexion, (B) eversion/inversion, and (C) internal/external rotation in children with (n = 20) and without (n = 10) flexible flatfoot during the stance phase (0-60% of the gait cycle) of comfortable walking. The standard deviations are shown in one direction.

**Figure 3:**
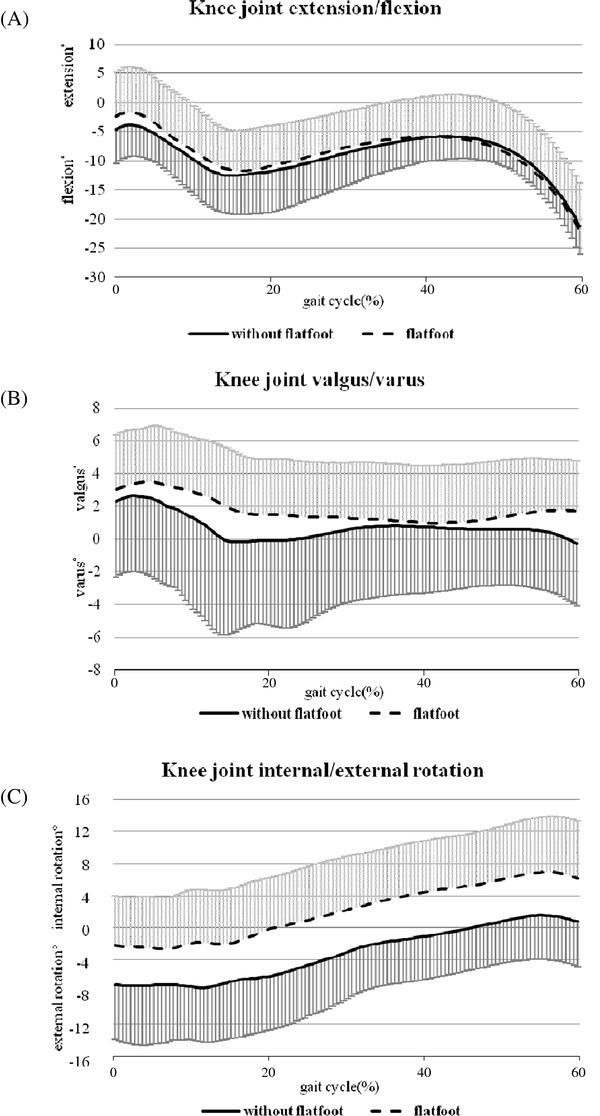
**Movement pattern of the knee joint**. (A) Extension/flexion, (B) valgus/varus, and (C) internal/external rotation in children with (n = 20) and without (n = 10) flexible flatfoot during the stance phase (0-60% of the gait cycle) of comfortable walking. The standard deviations are shown in one direction.

**Figure 4:**
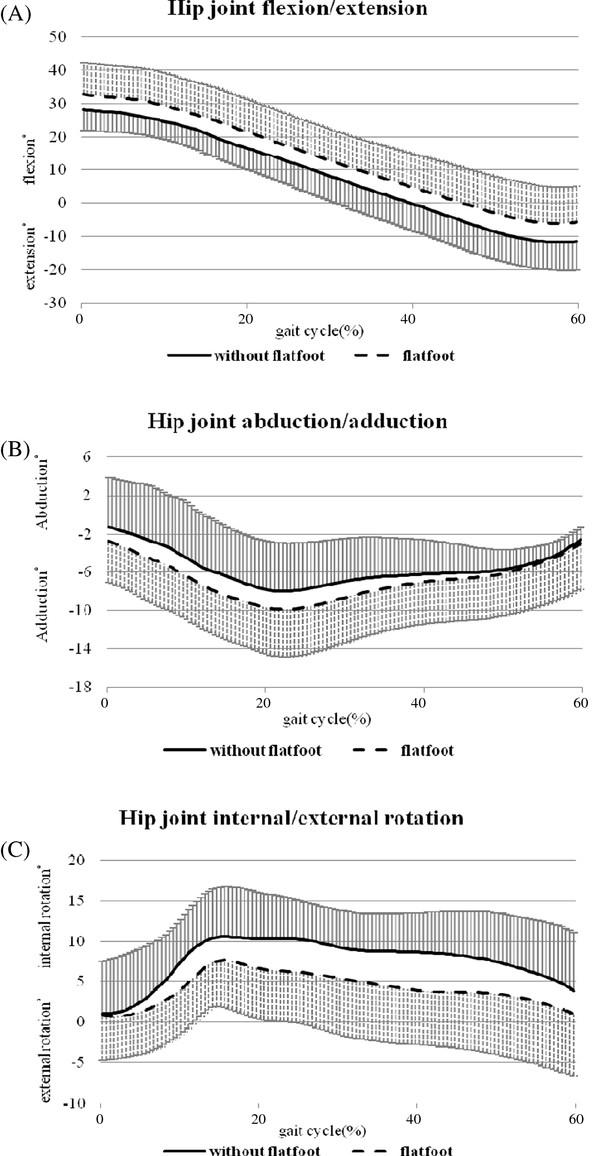
**Movement pattern of the hip joint**. (A) Flexion/extension, (B) abduction/adduction, and (C) internal/external rotation in children with (n = 20) and without (n = 10) flexible flatfoot during the stance phase (0-60% of the gait cycle) of comfortable walking. The standard deviations are shown in one direction.

The averaged angular displacements of the calcaneus, knee and hip joint during the stance phase of a gait cycle are summarized in Table 2. Children with or without flexible flatfoot exhibited similar patterns of movement around hip, knee and ankle joints (Figure 2, 3 and 4).

**Table 2:** Comparison of the kinematic parameters between children with and without flexible flatfoot^a^

Kinematic variables	Flexible Flatfoot group N = 20 Mean (SD)	Control group N = 10 Mean (SD)	Mean Difference (95% CI)
Calcaneal dorsi-flexion at heel contact (°)	-1.05 (6.16)	0.37 (6.06)	1.42 (-3.44 to 6.27)
Max. calcaneal dorsi-flexion (°)	7.61(5.91)	8.23(4.29)	6.28 (-3.69 to 4.94)
Max. calcaneal plantar-flexion (°)	8.14(5.43)	7.63(5.58)	5.11 (-3.84 to 4.86)
Total range of calcaneal dorsi-/plantar-flexion(°)	15.74(4.35)	15.86(5.2)	0.12 (-3.57 to 3.80)
Calcaneal eversion at heel contact (°)	-5.77 (3.61)	-5.17 (3.11)	0.60 (-2.14 to 3.35)
Max. calcaneal eversion (°)	3.24(2.85)	4.50(2.99)	1.26 (-1.03 to 3.56)
Max. calcaneal inversion (°)	7.69(4.01)	6.33(2.53)	1.36 (-1.50 to 4.21)
Total range of calcaneal eversion/inversion(°)	10.93(3.24)	10.83(3.44)	-0.96 (-2.72 to 2.52)
Calcaneal eversion excursion (°)	9.75(3.32)	10.27(3.55)	0.53 (-2.16 to 3.22)
Calcaneal internal rotation at heel contact (°)	-4.08 (9.53)	-0.75 (24.03)	7.89 (-14.15 to 20.81)
Max. calacaneal external rotation (°)	10.84(8.39)	5.94(23.81)	2.64 (-14.29 to 19.57)
Max. calacaneal internal rotation (°)	-0.41(8.59)	2.23(23.33)	4.90 (-12.36 to 22.15)
Total range of calcaneal external/internal rotation (°)	10.43(3.12)	8.17(4.02)	-2.26 (-4.98 to 4.70)
Knee joint extension at heel contact (°)	-2.10 (7.55)	-4.62 (5.78)	-2.52 (-7.67 to 2.62)
Max. knee joint flexion (°)	22.85(7.86)	22.12(5.30)	-3.03 (-7.98 to 1.92)
Max. knee joint extension (°)	1.38(6.78)	-1.65(4.92)	0.73 (-4.93 to 6.40)
Total range of knee joint flexion/extension (°)	24.24(6.03)	20.48(4.03)	-3.76 (-8.10 to 0.58)
Knee joint valgus at heel contact (°)	2.61 (3.47)	2.32 (4.63)	-0.29 (-3.37 to 2.79)
Max. knee joint valgus (°)	4.56(3.24)	3.42(4.89)	-1.14 (-4.20 to 1.90)
Max. knee joint varus (°)	0.22(3.16)	2.21(4.42)	-1.99 (-4.86 to 0.88)
Total range of knee joint valgus/varus (°)	4.78(1.67)	5.62(1.97)	0.84 (-0.56 to 2.25)
Knee joint internal rotation at heel contact (°)	-2.47 (5.89)	-7.09 (6.84)	-4.62 (-9.99 to 0.75)
Max. knee joint internal rotation (°)	7.94(6.05)	3.08(5.07)	**-4.86 (-9.70 to -0.01)**
Max. knee joint external rotation (°)	4.90(6.81)	10.18(6.54)	-5.28 (-10.62 to 0.06)
Total range of knee joint internal/external rotation (°)	12.84(3.90)	13.23(3.09)	0.42 (-2.49 to 3.32)
Knee joint internal rotation excursion	12.82(3.95)	12.37(4.66)	-0.45 (-3.78 to 2.87)
Hip joint flexion at heel contact (°)	31.49 (11.99)	28.30 (6.16)	-3.18 (-14.49 to 5.13)
Max. hip joint flexion (°)	33.3(9.39)	28.77(6.11)	-4.53 (-11.25 to 2.20)
Max. hip joint extension (°)	6.80(10.86)	12.24(8.38)	-5.44 (-13.47 to 2.59)
Total range of hip joint flexion/extension (°)	40.09(4.02)	41.01(4.39)	0.92 (-4.08 to 5.91)
Hip joint abduction at heel contact (°)	-2.25 (3.67)	-1.23 (5.15)	1.00 (-2.32 to 4.34)
Max. hip joint abduction (°)	-1.21(4.22)	0.42(3.52)	1.62 (-1.55 to 4.80)
Max. hip joint adduction (°)	10.54(4.86)	8.93(4.39)	1.61 (-2.13 to 5.35)
Total range of hip joint abduction/adduction (°)	9.33(2.61)	9.35(3.52)	0.01 (-2.31 to 2.34)
Hip joint internal rotation at heel contact (°)	0.75 (5.22)	1.10 (6.43)	0.35 (-4.11 to 4.82)
Max. hip joint internal rotation (°)	9.80(6.18)	13.53(5.05)	3.73 (-0.90 to 8.36)
Max. hip joint external rotation (°)	2.85(4.74)	0.55(6.55)	2.30 (-1.97 to 6.58)
Total range of hip joint internal/external rotation (°)	12.65(2.91)	14.08(3.77)	1.43 (-1.12 to 3.97)
Hip joint internal rotation excursion (°)	10.61(2.91)	13.33(3.86)	**2.71 (0.33 to 5.10)**

95%CI: 95% Confidence interval of the difference

Statistically significant associations are given in bold face

The results of ANOVA showed no significant main effect or significant interaction between group and other factors (joint, plane of movement, points across stance phase) (P > 0.05). Despite this, in order to examine the potential group differences, t-tests were used to compare all kinematic variables between two groups. The analyses revealed that children with flexible flatfeet tended to have a larger maximum knee internal rotation angle (mean difference [95% CI]: -4.86° [-9.70°, -0.01°], P = 0.049) and less hip internal rotation excursion (mean difference [95% CI]: 2.71° [0.33°, 5.10°], P = 0.027) in the stance phase (Table 2) as compared to the children with normal arches.

## Discussion

This study described and compared the three-dimensional kinematics of the calcaneus, knee, and hip joints in elementary school children with and without flexible flatfeet. During the stance phase of the gait, the pattern of calcaneal movement was similar between the two groups, and was in agreement with the pattern reported in previous kinematic studies [8,10,16,24-29]. The maximum dorsiflexion angle found in this study was around 8°, which was comparable to the data reported by Ganley and Powers found in adults [25]. With or without flexible flatfeet, children in our study showed a maximum calcaneal eversion angle of 3° to 4.5° during the stance phase. Levinger et al. reported a similar maximum calcaneal eversion angle in adults [8]. A larger calcaneal eversion angle between 10° to 15° was observed in both adults and children with flatfeet [15,29] and with normal arch [26,29]. Differences in data collection methods and joint angle definitions would partly contribute to these discrepancies.

Excessive calcaneal eversion is suggested as an important factor related to lower extremity kinematic deviations in subjects with flexible flatfeet [8,9,26]. Despite children of the flexible flatfoot group showed a significantly larger calcaneal eversion angle in quiet standing (Table 1), similar calcaneal movement during walking was noted for both groups (Table 2). The discrepancies in calcaneal angular changes during quiet standing and walking might be a result of different loading conditions (relaxed standing with most muscles quiet vs. walking with active muscle control) and diverse coupling movement patterns of the whole lower extremity kinematic chain.

Our results were supported by findings of Twomey et al. that kinematic differences in the foot between children with normal and low arched feet were small [29]. In adults, Hunt and Smith found no statistical difference in the total range of calcaneal frontal plane movement between normal and flexible flatfoot groups [30]; whereas Kernozek and Ricard [31], and Williams et al. [32] demonstrated that a lower arch height led to greater calcaneal eversion excursion and maximum calcaneal eversion angle. Levinger and colleagues also identified a trend of larger rearfoot eversion in adults with flexible flatfeet [8]. These inconsistent findings might be a result of subject variations, such as different age groups and severity of flatfoot, and differences in the definition and classification of flatfoot, and in methods of motion measurement [8,29-32]. In addition, researchers should not overlook the contribution of midfoot and forefoot joints to the presence of flexible flatfoot [1,29,33]. The collapsed medial arch during weight bearing could also be a result of a pronated midfoot or forefoot, which was not measured as part of this study.

With the tight connection between tibia and talus under a weight bearing condition, it has been proposed that the presentation of flexible flatfoot could be associated with abnormal knee and/or hip rotation in the transverse plane, which might lead to lower extremity problems, such as patellofemoral pain [34]. Despite similar sagittal and frontal plane knee joint movement in the two groups, we found that children with flexible flatfoot exhibited a trend towards increased knee internal rotation in the transverse plane throughout the stance phase (Figure 3(C), Table 2). Therefore, possible future injuries associated with this alteration should be carefully monitored in this population.

No previous studies have compared hip joint kinematics between subjects with and without flatfoot conditions. This is the first study to identify that children with flexible flatfeet have a tendency towards greater hip flexion, adduction and less hip internal rotation during the stance phase of gait (Figure 4). At the instance of heel strike, the hip exhibited a similar rotational position in the transverse plane for both groups of subjects; as the gait progressed, children with flexible flatfeet demonstrated less overall movement of internal hip rotation during the weight bearing phase. The altered knee and hip kinematics might be a compensatory mechanism for excessive knee joint internal rotation. Whether changes of hip and knee kinematic performance were unique in our subject group, and whether these alterations linked to any clinical symptoms should be examined further.

There were a few limitations of this study. We used a skin-based method of sensor placement, which usually causes minor measurement errors because of the skin movement. These measurement errors might contribute to larger SEMs for some parameters such as maximum calcaneal eversion, thus resulting in our insignificant findings. Although the measurement method including the kinematic model used in this study, has been reported previously in adults, it has not been validated in children. Whether or not factors such as different anthropometric proportions in children jeopardized our measurement accuracy should be considered. The screening method for flexible flatfoot in this study was clinical observation combined with the measurement of navicular height. Although this method is not as accurate as the imaging techniques such as radiography, it is practical and commonly used in clinical practice. Lastly, the alignment and movement of the midfoot and forefoot, which could possibly impact our findings, were not measured in this study. An investigation taking account of midfoot and forefoot structure may need to be considered in future studies.

## Conclusions

Although children with flexible flatfoot demonstrated a larger calcaneal eversion while standing, no kinematic adaptation during walking was noted in the flexible flatfoot group. More evidence is needed before any clinical implication could be drawn. We suggested that future research should recruit more subjects and take the influence of the mid-foot and forefoot into consideration when examining lower extremity kinematics in children with flexible flatfoot.

## Abbreviations

Q angle: Quadriceps angle; SD: Standard deviation; 95% CI: 95% confidence interval; ICC: Intraclass correlation coefficient; SEM: Standard error of measurement; ANOVA: Analysis of variance.

## Competing interests

The authors confirm that there are no known conflicts of interest associated with this publication and there has been no significant financial support for this work that could have influenced its outcome.

## Authors' contributions

YF Shih and CY Chen designed the study. CY Chen performed data acquisition. CY Chen, YF Shih and HC Lin analyzed and interpreted the data. YF Shih and CY Chen wrote the manuscript. YF Shih, CY Chen, HC Lin, and WY Chen read and approved the manuscript.

## Pre-publication history

The pre-publication history for this paper can be accessed here:

http://www.biomedcentral.com/1471-2474/13/31/prepub
